# Lesion Penetration and Activity Limit the Utility of Second-Line Injectable Agents in Pulmonary Tuberculosis

**DOI:** 10.1128/AAC.00506-21

**Published:** 2021-09-17

**Authors:** Jacqueline P. Ernest, Jansy Sarathy, Ning Wang, Firat Kaya, Matthew D. Zimmerman, Natasha Strydom, Han Wang, Min Xie, Martin Gengenbacher, Laura E. Via, Clifton E. Barry, Claire L. Carter, Radojka M. Savic, Véronique Dartois

**Affiliations:** a Department of Bioengineering and Therapeutic Sciences, University of California, San Francisco, San Francisco, California, USA; b Center for Discovery and Innovation, Hackensack Meridian Health, Nutley, New Jersey, USA; c Tuberculosis Research Section, Laboratory of Clinical Immunology and Microbiology, NIAID, NIH, Bethesda, Maryland, USA; d Institute of Infectious Disease and Molecular Medicine, University of Cape Town, Observatory, South Africa; e Hackensack School of Medicine, Hackensack Meridian Health, Nutley, New Jersey, USA

**Keywords:** aminoglycoside, drug tolerance, multidrug resistant, pharmacokinetics, tissue penetration, tuberculosis

## Abstract

Amikacin and kanamycin are second-line injectables used in the treatment of multidrug-resistant tuberculosis (MDR-TB) based on the clinical utility of streptomycin, another aminoglycoside and first-line anti-TB drug. While streptomycin was tested as a single agent in the first controlled TB clinical trial, introduction of amikacin and kanamycin into MDR-TB regimens was not preceded by randomized controlled trials. A recent large retrospective meta-analysis revealed that compared with regimens without any injectable drug, amikacin provided modest benefits, and kanamycin was associated with worse outcomes. Although their long-term use can cause irreversible ototoxicity, they remain part of MDR-TB regimens because they have a role in preventing emergence of resistance to other drugs. To quantify the contribution of amikacin and kanamycin to second-line regimens, we applied two-dimensional matrix-assisted laser desorption ionization (MALDI) mass spectrometry imaging in large lung lesions, quantified drug exposure in lung and in lesions of rabbits with active TB, and measured the concentrations required to kill or inhibit growth of the resident bacterial populations. Using these metrics, we applied site-of-action pharmacokinetic and pharmacodynamic (PK-PD) concepts and simulated drug coverage in patients’ lung lesions. The results provide a pharmacological explanation for the limited clinical utility of both agents and reveal better PK-PD lesion coverage for amikacin than kanamycin, consistent with retrospective data of contribution to treatment success. Together with recent mechanistic studies dissecting antibacterial activity from aminoglycoside ototoxicity, the limited but rapid penetration of streptomycin, amikacin, and kanamycin to the sites of TB disease supports the development of analogs with improved efficacy and tolerability.

## TEXT

Kanamycin (KAN) and amikacin (AMK) are injectable aminoglycoside antibiotics discovered in the 1950s to 1970s and were among the first agents approved to treat Gram-negative and Gram-positive bacterial infections (1). They were repurposed to treat multidrug-resistant tuberculosis (MDR-TB) based on the clinical utility of streptomycin (SM), an earlier injectable aminoglycoside tested as a single agent in the first controlled clinical trial with TB patients in 1946 (2). In recent years, however, large retrospective studies and meta-analyses provided limited evidence that use of second-line injectable aminoglycosides KAN and AMK is associated with an increased likelihood of treatment success (3, 4). Consistent with these observations, unfavorable outcomes were similar in patients susceptible versus resistant to KAN and AMK (5). Prospective observational studies and randomized placebo-controlled trials were lacking at the time of their introduction in MDR-TB regimens. The results of early bactericidal activity (EBA) trials with AMK and liposomal AMK were published in 2001, showing barely detectable and negligible effects, respectively (6–8).

Second-line injectables can cause serious and irreversible ototoxicity, resulting in permanent hearing loss in 3% to more than 60% of the patients across studies (4, 9–12). This comes in addition to the logistical challenge and pain associated with daily injections for many months. Since the risks of severe ototoxic and nephrotoxic reactions are sharply increased in patients who receive prolonged therapy (12), aminoglycosides are only recommended for short-term treatment of severe infections not to exceed 7 to 10 days, with the exception of TB and nontuberculous mycobacterial infections.

Given the limited evidence of clinical utility and irreversible side effects, why were KAN and AMK introduced and kept in MDR-TB regimens? SM was the first antibiotic approved to treat TB and remained in use as a first-line agent in resource-limited countries until 2019 (13). Since KAN and AMK exhibit *in vitro* potency 4-fold and 2-fold lower than SM against Mycobacterium tuberculosis (Mtb), respectively (14), it was assumed they may achieve similar or minimally reduced efficacy. In mice and guinea pigs, high doses of AMK and KAN provided limited efficacy compared to SM and isoniazid (15). Efficacy was slightly improved when animals received high infectious doses via the intravenous or intracardial route and when treatment started immediately for up to 90 days (16, 17), and treatment was improved in γ-interferon (IFN) knockout mice (18). When AMK and KAN were tested side by side, AMK appeared more efficacious at comparable doses (17). Intrapulmonary delivery did not provide any benefit compared to subcutaneous injection of AMK (19). Liposomal formulation of AMK showed improved activity compared to free AMK in mice during the acute phase (20), which has been attributed to the liposomes favoring drug penetration into macrophages and retarding its clearance from the site of action (21). The predictive value of these efficacy studies is limited by the lack of a pharmacological rationale to select a dose that reproduces the pharmacokinetic-pharmacodynamic (PK-PD) target of aminoglycosides in TB patients (8, 22). While peak plasma concentration relative to MIC (*C*_max_/MIC) is considered the PK-PD driver of efficacy for aminoglycosides against most bacterial infections (23), no systematic *in vivo* studies have been conducted to confirm this for TB and establish PK-PD targets of efficacy (8). Based on PK-PD threshold of efficacy versus toxicity in patients (24), dose fractionation studies in the hollow fiber system (25), and PK-PD targets in other bacterial infections (26), the WHO recommends doses that achieve *C*_max_/MIC values of 10 for KAN and AMK (27, 28). Because most animal efficacy studies described above were performed in the “pre-PK” era, there were no PK data reported to assess PK-PD parameters. However, PK data from other studies (reference [29] and our unpublished results) indicate that *C*_max_/MIC must have been in excess of the WHO-recommended target and may have exceeded KAN and AMK exposures that are tolerated in humans. Despite the disappointing EBA trials, AMK and KAN were kept in MDR-TB regimens because they were believed to have a role in preventing emergence of resistance to other drugs (6). They are therefore considered “companion drugs” used to avert treatment failure due to acquired resistance against core drugs (30–32).

To provide a pharmacological rationale for the limited contribution of AMK and KAN to second-line regimens, we applied site-of-disease PK-PD concepts and measured drug exposure at the site of infection in rabbits with active TB relative to concentrations required to kill or inhibit growth of the resident bacterial populations. Using these metrics for assessing lesion PK-PD coverage in patients, we provide a pharmacological explanation for the limited clinical utility of both agents and the slightly more favorable contribution of AMK to MDR-TB regimens in retrospective studies compared to KAN.

## RESULTS

### AMK and KAN penetrate major TB lesion compartments to similar extents.

To build a translational model of lesion penetration for second-line injectables AMK and KAN, we first identified a rabbit dose that achieves exposure comparable to that of TB patients receiving a 1,000 mg intramuscular dose (33–38). The AMK concentration time profile was established in naive (uninfected) rabbits following a single intramuscular 60 mg/kg dose (Fig. S1A in the supplemental material). The ratio between peak plasma concentration (*C*_max_) and MIC is considered the primary PK-PD parameter driving antibacterial effects of the aminoglycosides (39), although a recent review and reappraisal of available literature and updated guidelines suggest that the area under the concentration-time curve (AUC)/MIC ratio may be a more reliable indicator of bacterial killing and clinical efficacy for these agents (40, 41). Overall, rabbits clear AMK faster than humans, leading to higher *C*_max_ values at human-equivalent AUC. To achieve a compromise between matching human *C*_max_ and human AUC, a dose of 25 mg/kg was selected and given daily for 3 days to reach steady state (Fig. S1 and Table S1). Since AMK and KAN display similar PK profiles and exposure in patients (26, 42, 43) and in rabbits (33, 44–46), the same dose of 25 mg/kg was selected for both agents. Next, the PK profile of AMK and KAN was obtained in TB-infected rabbits following three daily doses of 25 mg/kg, confirming identical exposure of AMK and KAN and reaching a compromise between matching *C*_max_ and AUC of TB patients receiving a daily dose of 1,000 mg (Fig. S2 and Table S1).

**FIG 1:**
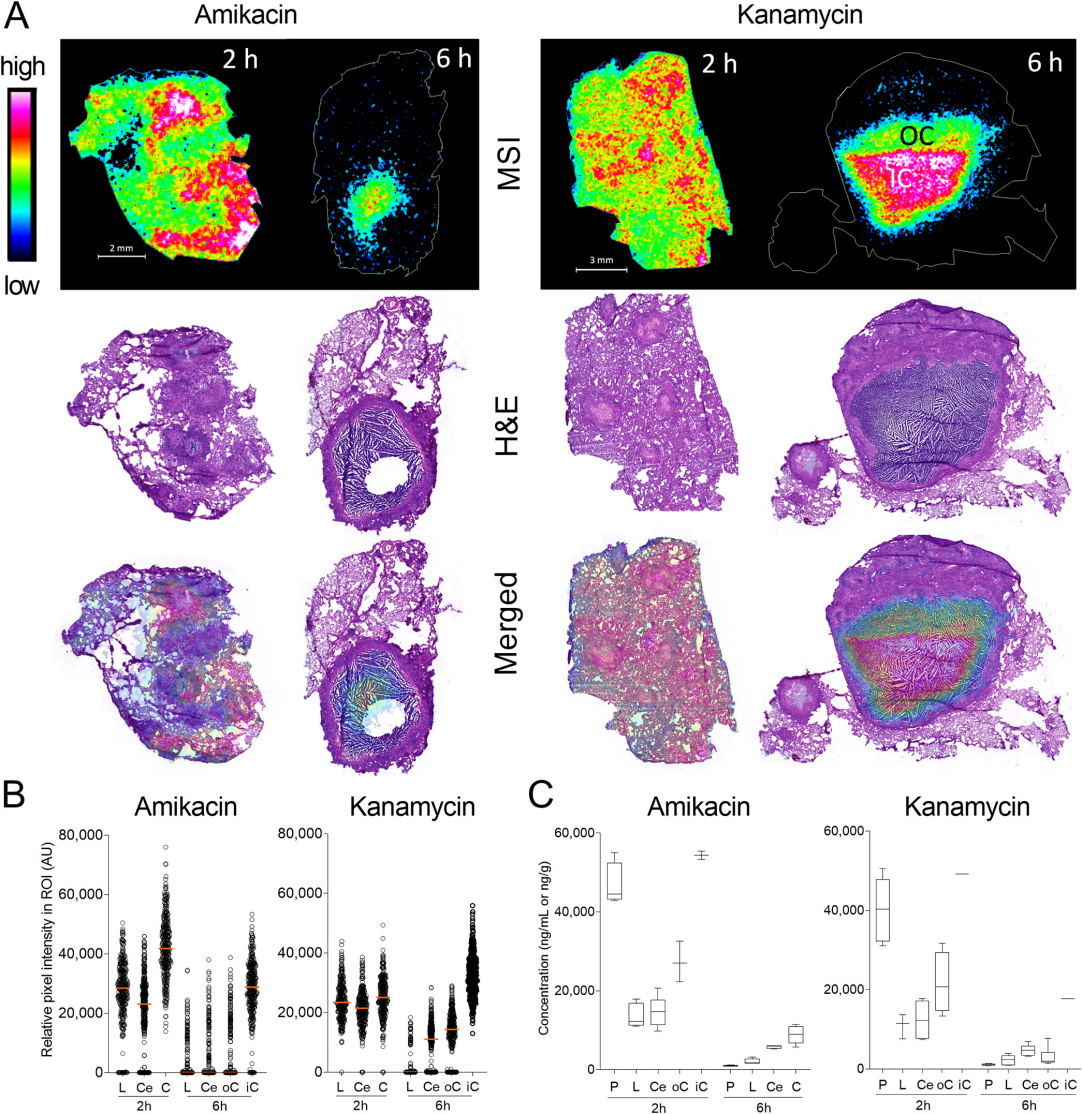
Distribution of KAN and AMK in lung, necrotic lesions, and cavities. (A) MALDI mass spectrometry (MS) ion maps of AMK and KAN in representative rabbit lung tissues collected 2 h and 6 h postdose at steady state. MS images of AMK and KAN distribution are displayed on the top. The hematoxylin and eosin (H&E) stains of the sections used for MSI are shown in the middle, and merged H&E and MS images are on the bottom; OC, outer caseum; IC, inner caseum. (B) Scatterplots of relative pixel (50 × 50 mm) intensities within regions of interest (ROI) drawn to encompass approximately 350 pixels within lung and lesion compartments as indicated; L, uninvolved lung; Ce, cellular rim; oC, outer caseum; iC, inner caseum. (C) Absolute concentrations of AMK and KAN in plasma and infected lung regions determined by laser-capture microdissection and LC/MS-MS; P, plasma; L, uninvolved lung; Ce, cellular rim; C, caseum; oC, outer caseum; iC, inner caseum.

To visualize the partitioning of AMK and KAN in necrotic lesions and surrounding lung tissue, we generated drug heat maps using matrix-assisted laser desorption ionization (MALDI) mass spectrometry imaging (MSI) in thin tissue sections collected 2 h and 6 h after the third dose (Fig. 1A). Sections imaged by MALDI MSI were washed and subsequently stained with hematoxylin and eosin (H&E) to reveal the underlying lesion structure and cellular composition. At 2 h postdose, penetration of both drugs was homogeneous throughout uninvolved lung and cellular and necrotic lesion compartments, with apparent higher abundance in denser tissue areas. Given the rapid clearance of aminoglycosides, plasma levels had fallen below 1 μg/ml at 6 h postdose (Fig. S1A). This was reflected by the low drug abundance in uninvolved lung and cellular lesion areas, which are well vascularized. In contrast, AMK and KAN were partially retained within caseous foci at 6 h, leading to highest signal intensity in the center of the necrotic cores. Lesions collected at 6 h postdose are larger than at 2 h due to interanimal variability in pathology. To obtain semiquantitative data from these drug ion maps, individual pixel intensities were plotted in regions of interest (ROIs) that were manually delineated based on immunopathology staining by H&E (uninvolved lung, cellular rim, and caseum). Outer and inner caseum were sampled separately when large necrotic lesions were present. The data confirmed the partitioning of AMK and KAN visualized by MALDI MSI (Fig. 1B). To measure absolute drug concentrations in defined lung and lesion areas, we collected samples in adjacent tissue sections by laser-capture microdissection (LCM) (47) in 5 to 9 lesions per drug at 2 h and 6 h postdose. AMK and KAN concentrations were measured in microdissected areas and concomitantly collected plasma by conventional mass spectrometry. As anticipated based on MALDI MSI images and pixel intensities, we found higher AMK and KAN concentrations in caseum than in cellular lesion rims. Both drugs were higher in plasma than in tissues at 2 h postdose, while the opposite was observed at 6 h. Absolute drug levels decreased in all compartments between 2 h and 6 h postdose (Fig. 1C; see also Fig. S3).

**FIG 2:**
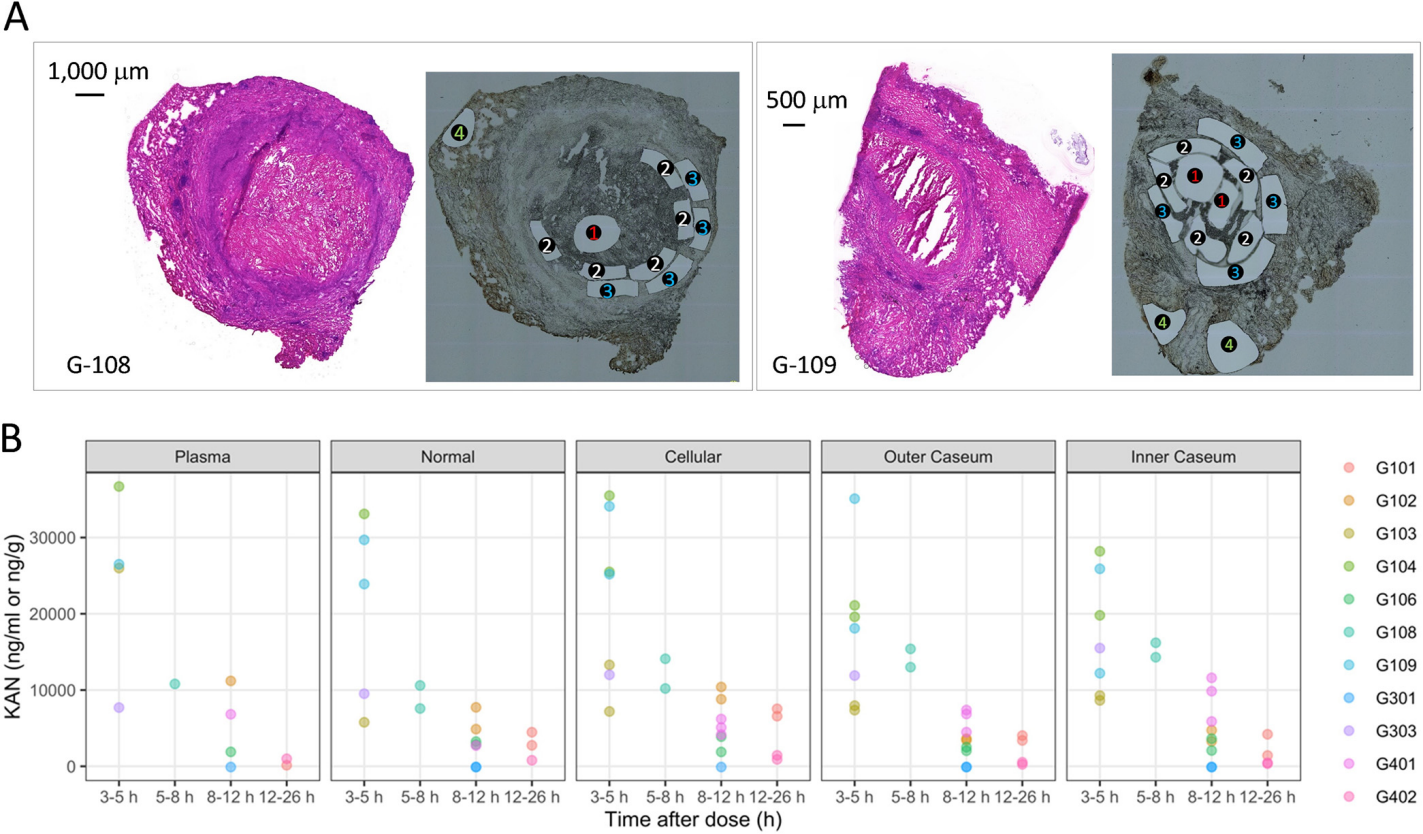
KAN distribution in human lung lesions. (A) Typical examples of histology staining and laser-capture microdissection (LCM) of thin human lesion sections. Two large necrotic lesions were collected from the resected lung tissue of human subjects G-108 and G-109. Adjacent lesion sections were used for hematoxylin and eosin (H&E) staining (left) to guide LCM sample collection (right); 1, inner caseum; 2, outer caseum; 3, cellular rim; 4, uninvolved lung. Laser-dissected pieces belonging to the same tissue compartment were pooled for quantitation by LC-MS/MS. (B) Concentrations of KAN in plasma and 20 resected human lesions from 10 subjects, collected at various times from 3 h to 26 h after a single dose or multiple doses of 1,000 mg of KAN injected intramuscularly (49). Concentrations were determined in thin section samples collected by LCM and analyzed by LC/MS-MS (47). Each color corresponds to one subject. Raw data are provided in Data Set S1 in the supplemental material.

To complement the rabbit data set and determine whether the partitioning of KAN between uninvolved lung, cellular, and caseous rabbit lesion compartments extends to humans, we applied LCM to 20 large necrotic lesions and cavities collected in a previous clinical study from 10 subjects who had received 1,000 mg of KAN by intramuscular injection (48, 49). TB patients who undergo lung resection usually present with drug refractory cavitary disease, thus enabling lesion PK investigations in a wider spectrum of lesion type and size (48). These subjects had MDR-TB or extensively drug-resistant tuberculosis (XDR-TB) and either received KAN as part of their optimized drug regimen or received a single dose of KAN in addition to their background regimen on the day of scheduled lung resection (Table S2). Although within-subject and across-subject variability was higher than in rabbits, as expected, we observed similarly rapid diffusion into caseum in all subjects both following a single dose and at steady state (Fig. 2; Data Set S1). KAN concentrations were higher in caseum than surrounding cellular and lung tissue in a minority of lesions, which did not appear to be associated with steady state (Table S2 and Data Set S1). There was no trend of increased partitioning into caseum relative to the surrounding tissue at steady state, regardless of the time point postdose. Overall, KAN concentrations decreased rapidly over the course of the dosing interval, as seen in rabbits.

To build a translational model of AMK and KAN penetration at the site of TB disease, we measured drug concentrations in serial blood samples and whole lung and lesion homogenates in groups of 3 TB-infected rabbits dosed with 25 mg/kg of AMK or KAN and analyzed at 2 h and 6 h after the third daily dose. The total number of observations and the concentrations of AMK and KAN data in plasma, uninvolved lung, and cellular and necrotic lesions are shown in Table S3 and Fig. S1B.

**FIG 3:**
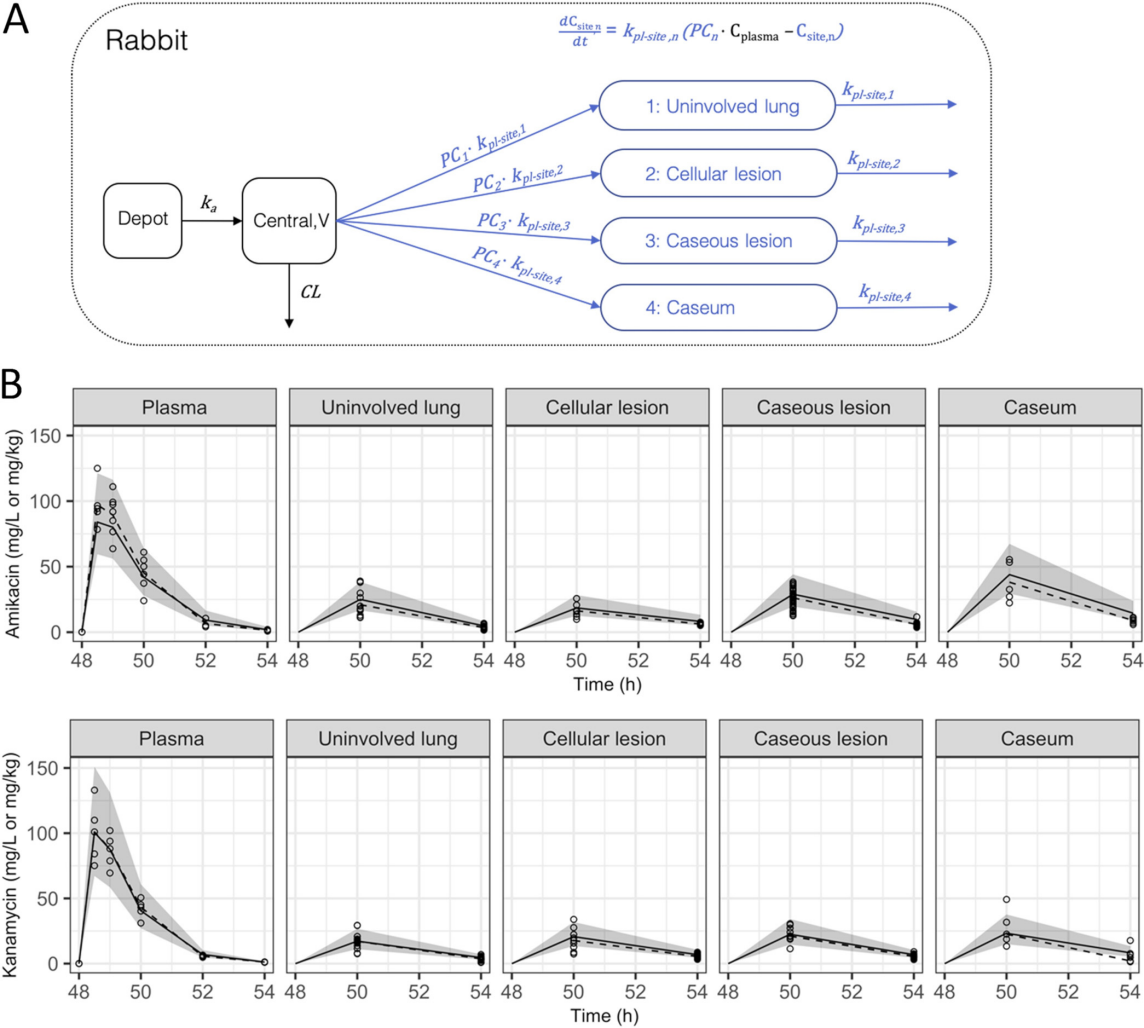
Model structure and visual predictive checks (VPC) for plasma-to-tissue distribution of AMK and KAN. (A) Model structure for the rabbit plasma and site-of-action PK. (B) Scatter VPC of rabbit plasma-to-tissue model for AMK and KAN. Points and dashed lines represent observed data points and median of observed data, respectively. Shaded area and solid lines represent the 90% prediction interval and the median of 1,000 simulations, respectively; *k*_a_, rate of absorption; V, central volume of distribution; CL, clearance; PC, partition coefficient or the ratio of drug at site compared to plasma; *k*_pl-site_, distribution rate constant; *C*_plasma_, concentration in plasma; *C*_site_, concentration at site of action.

KAN plasma PKs in rabbits were best described by a one-compartment model with interindividual variability on bioavailability. Clearance (CL) was estimated to be 0.45 liters/h, and volume of distribution (V) was estimated to be 0.5 liters, indicating rapid elimination (elimination half-life of 47 min). Bioavailability was fixed to 1, while the rate of absorption after intramuscular injection was estimated to be 2.94 (absorption half-life of 14 min). A slope-intercept model best described the residual error, with a proportional error of 9.23% and additive error of 0.034 mg/liter. The model structure of rabbit plasma and site-of-action PK and scatter visual predictive check of AMK and KAN distribution from plasma to infected lung tissues are shown in Fig. 3. KAN exposure was greater in plasma than in any other tissue compartment. Estimated plasma-to-lesion partition coefficients were 0.338, 0.454, 0.476, and 0.497 for uninvolved lung, cellular lesions, caseous lesions, and caseum, respectively, indicating that all lesion compartments see AMK and KAN exposure less than half the exposure measured in plasma. Of the tissue compartments, caseum had the highest exposure followed by caseous lesions, cellular lesions, and uninvolved lung. The residual error in tissue compartments was best described by a proportional error model. Final model parameters are listed in Table 1.

**TABLE 1:** Final parameter estimates for the rabbit plasma-to-lesion PK model

Compartment	Parameter^*a*^	AMK Value, RSE (%)	KAN Value, RSE (%)
Plasma	*k*_a_ (1/h)	2.51 (14.7)	2.94 (10.2)
	CL (liter/h)	0.461 (8.0)	0.447 (9.8)
	V (liter)	0.572 (8.3)	0.502 (9.8)
	F1	1 FIX	1 FIX
	IIV F1 (CV %)	19.8 (23.3)	21.5 (10.1)
	IIV CL (CV %)	10.4 (18.5)	
	Proportional error (CV %)	16.4 (17.2)	9.23 (11.6)
	Additive error (mg/liter)	0.0568 (23.1)	0.0335 (22.0)
Uninvolved lung	Rate (*k*_pl-site,1_) (1/h)	0.716 (5.8)	0.493 (10.3)
	Partition coefficient (PC_1_)	0.437 (5.0)	0.338 (3.1)
	Proportional error (CV %)	37.8 (10.6)	44.5 (21.0)
Cellular lesions	Rate (*k*_pl-site,2_) (1/h)	0.385 (10.0)	0.419 (9.0)
	Partition coefficient (PC_2_)	0.462 (7.3)	0.454 (6.1)
	Proportional error (CV %)	25.0 (13.4)	34.7 (7.1)
Caseous lesions	Rate (*k*_pl-site,3_) (1/h)	0.490 (7.9)	0.448 (15.1)
	Partition coefficient (PC_3_)	0.618 (6.7)	0.476 (9.9)
	Proportional error (CV %)	29.6 (8.2)	32.7 (12.8)
Caseum	Rate (*k*_pl-site,4_) (1/h)	0.496 (8.1)	0.395 (22.6)
	Partition coefficient (PC_4_)	0.927 (8.0)	0.497 (21.3)
	Proportional error (CV %)	31.5 (14.8)	87.9 (18.6)

a *k*_a_, rate of absorption; 1/h, per hour; CL, clearance; V, central volume of distribution; F1, bioavailability; 1 FIX, parameter was fixed to 1; IIV, interindividual variability; CV, coefficient of variance; RSE, relative standard error; *k*_pl-site_, distribution rate constant (see Fig. 2A for rate constant and partition coefficient description).

AMK plasma PKs were similarly described by a one-compartment model with interindividual variability on bioavailability and CL. CL and V were estimated to be 0.461 liters/h and 0.572 liters (elimination half-life of 52 min). Bioavailability was fixed to 1, while the rate of absorption after intramuscular injection was estimated to be 2.51 (absorption half-life of 17 min). A slope-intercept model best described the residual error, with a proportional error of 16.4% and additive error of 0.057 mg/liter. AMK exposure was greater in plasma than in any other tissue compartment. Plasma-to-lesion partitioning was 0.437, 0.462, 0.618, and 0.927 for uninvolved lung, cellular lesions, caseous lesions, and caseum, respectively, indicating highest exposure in caseum (Table 1). Overall, KAN and AMK presented similar plasma PK profiles and showed modest but comparable penetration at all sites of pulmonary disease, with higher partitioning in caseum than in other lung areas. Interestingly, all partition coefficients were greater for AMK than KAN in the lung and in lesions, particularly in caseum. A similar trend was observed in a limited data set of streptomycin (SM) distribution in rabbit lung and lesions, showing lung-to-plasma and cellular lesion-to-plasma concentration ratios ranging from 0.3 to 0.5 and cavity caseum-to-plasma concentration ratios of 1.0 to 1.5 (Fig. S4). Thus, the three injectable aminoglycosides used in the treatment of TB have similar distribution patterns into lung lesions.

To further understand the higher retention of AMK, KAN, and SM in caseum than in vascularized cellular compartments, we measured the nonspecific binding of AMK, KAN, and SM in *ex vivo* caseum and found moderate to high binding and free fractions ranging from 4% to 10% (caseum *f*_u_) for all three drugs, in stark contrast with their low protein binding and high unbound fraction in plasma (plasma *f*_u_) reported in the literature (Table 2). This pattern is unique to the aminoglycosides and is consistent with rapid distribution from plasma to caseum and slightly prolonged retention in caseum given the low plasma and high caseum binding. In contrast, most TB drugs exhibit only slightly higher nonspecific binding in caseum than in plasma (50) and achieve higher concentrations in cellular than in necrotic and caseous lesion areas. To determine what contributes to their rapid and moderate distribution in cellular lesion areas, we measured the uptake of AMK, KAN, and SM in THP-1-derived macrophages *in vitro*. We found intracellular-to-extracellular concentration ratios between 2 and 3, similar to linezolid and falling in the “low uptake” category compared to other TB drugs (Table 2) (48, 51, 52). Thus, the high caseum binding and modest uptake in macrophages is consistent with the partitioning patterns of aminoglycosides in the cellular and necrotic regions of TB lesions.

**TABLE 2:** *In vitro* lesion PK properties of the aminoglycosides

Property^*a*^	AMK	KAN	SM	BDQ	MXF	LZD	References
IC/EC ratio in THP-1 macrophages	3.6 ± 0.2	3.8 ± 2.9	2.1 ± 0.7	176.5 ± 164.4	8.8 ± 4.5	2.3 ± 0.9	This work
Caseum *f*_u_ (%)	3.6 ± 0.3	4.8 ± 1.4	10.5 ± 1.9	<0.01	16.8 ± 1.8	27.9 ± 2.2	This work
Plasma *f*_u_ (%)	>90	∼100	65	<0.1	50–60	70	73, 75

a IC/EC, intracellular to extracellular concentration ratio after 30 min of incubation; *f*_u_, fraction unbound; BDQ, bedaquiline; MXF, moxifloxacin; LZD, linezolid.

### AMK and KAN are weakly active against M. tuberculosis populations found in lesions.

**FIG 4:**
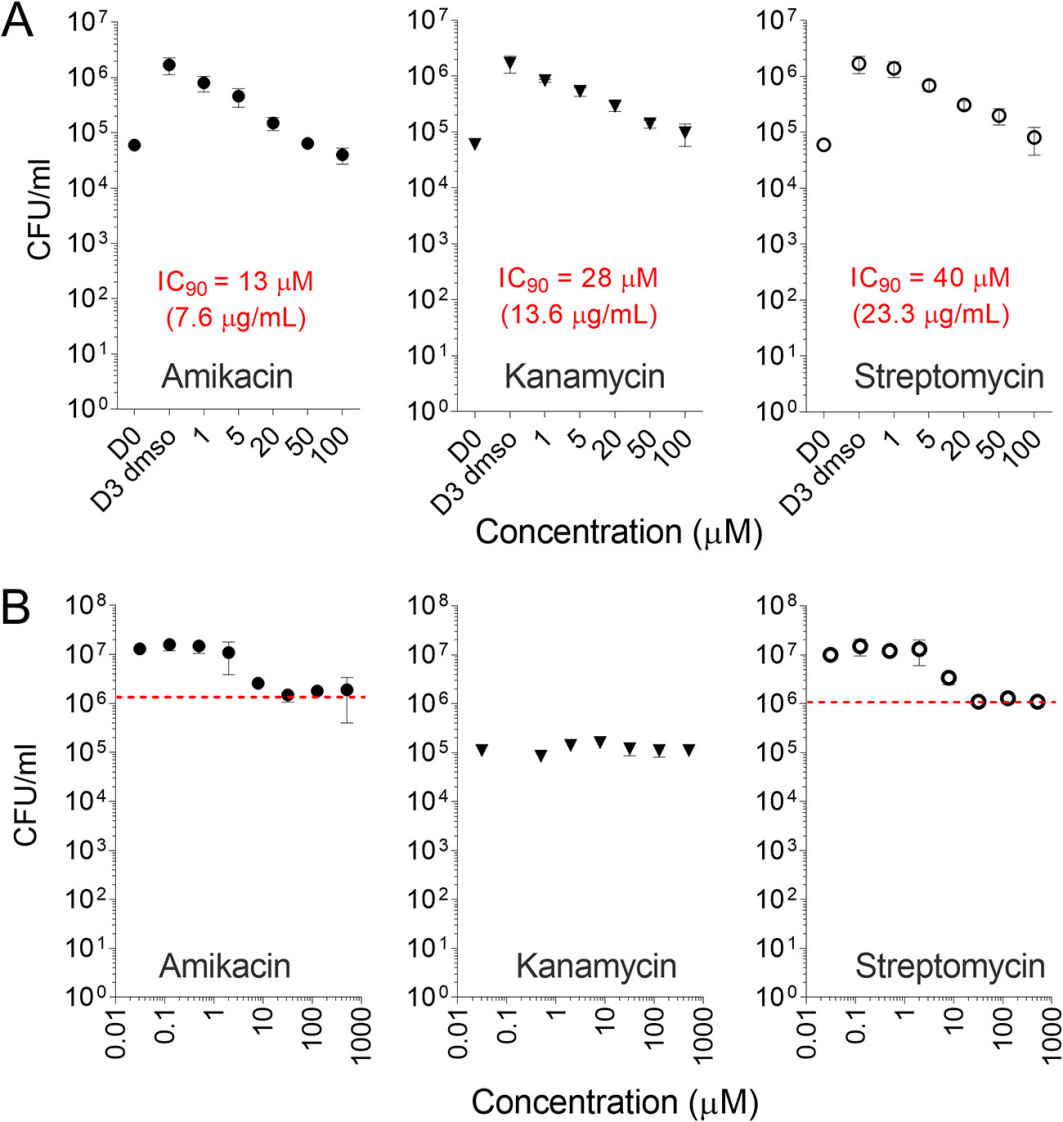
Potency of aminoglycosides against typical Mtb subpopulations found in lesions. (A) Aminoglycoside growth inhibitory activity against intracellular Mtb in THP-1-derived macrophages. Intracellular bacterial burden is shown for treated and drug-free control samples after 3 days of incubation at the concentrations indicated; D0, bacterial burden before drug treatment; D3 dmso, drug-free control on day 3. The experiment was performed twice with technical triplicates; one representative experiment is shown. (B) Bactericidal activity of aminoglycosides against nonreplicating Mtb persisters in *ex vivo* rabbit caseum (53). The red dotted line indicates the one-log kill mark or caseum MBC_90_.

The MIC values of AMK, KAN, and SM against a panel of clinical Mtb isolates have been reported in the literature. MIC distributions against a large panel of susceptible isolates center around 0.5 mg/liter for SM, 1 mg/liter for AMK, and 2 mg/liter for KAN (14). To place the lesion concentrations of AMK and KAN into pharmacodynamic context at the site of disease, we measured (i) the concentrations required for growth inhibition and killing of intracellular Mtb in macrophages and (ii) the concentrations required to kill nonreplicating Mtb in *ex vivo* caseum (53). SM was included in all assays as a first-line reference aminoglycoside tested as a single agent in early clinical trials (54). In infected THP-1-derived macrophages treated for 3 days, 90% growth inhibition of intracellular Mtb was achieved between 13 and 40 μM or 7.6, 13.6, and 23.3 mg/liter for AMK, KAN, and SM, respectively. No bacterial killing was observed up to 100 μM; all three drugs exerted a static effect only (Fig. 4A). Against nonreplicating persisters in caseum, both AMK and SM achieved a 1-log kill around 32 μM (19 mg/liter). KAN was inactive up to 512 μM (Fig. 4B). Overall, potency was low against intracellular and nonreplicating Mtb compared to standard MIC values, and KAN was less potent than AMK, consistent with reported MIC and minimal bactericidal concentration (MBC) values (Table 3).

**TABLE 3:** Summary of *in vitro* potency of the aminoglycosides in relevant assays

Potency assay^*a*^	AMK	KAN	SM	Reference
MIC (mg/liter)	1	2	0.5	14, 76
MBC (mg/liter)	1.0–8.0	3.0–32.0	0.5–8.0	76, 77
MacIC_90_ (mg/liter)	8	13	40	This work
casMBC_90_ (mg/liter)	19	>248	19	This work

a MacIC_90_, concentration at which 90% of bacterial growth is inhibited in THP-1-derived macrophages; casMBC_90_, concentration at which 90% of bacteria are killed in *ex vivo* caseum.

### Simulation of lesion pharmacokinetics-pharmacodynamics reveals poor lesion coverage.

Aminoglycosides exert concentration-dependent activity *in vivo*, with both *C*_max_/MIC and AUC/MIC driving antibacterial effect (39–41). While these PK-PD drivers were established for bacterial infections other than TB, they are generally recognized as a property of the class. The long postantibiotic effect of aminoglycosides provides a rational explanation for *C*_max_/MIC-driven killing (26). Given the limited evidence that use of KAN and AMK is associated with an increased likelihood of treatment success (3, 4, 55), we hypothesized that lesion-centric PK-PD parameters are better predictors of efficacy than conventional plasma *C*_max_/MIC or AUC/MIC, as observed for other TB drugs (56, 57).

**FIG 5:**
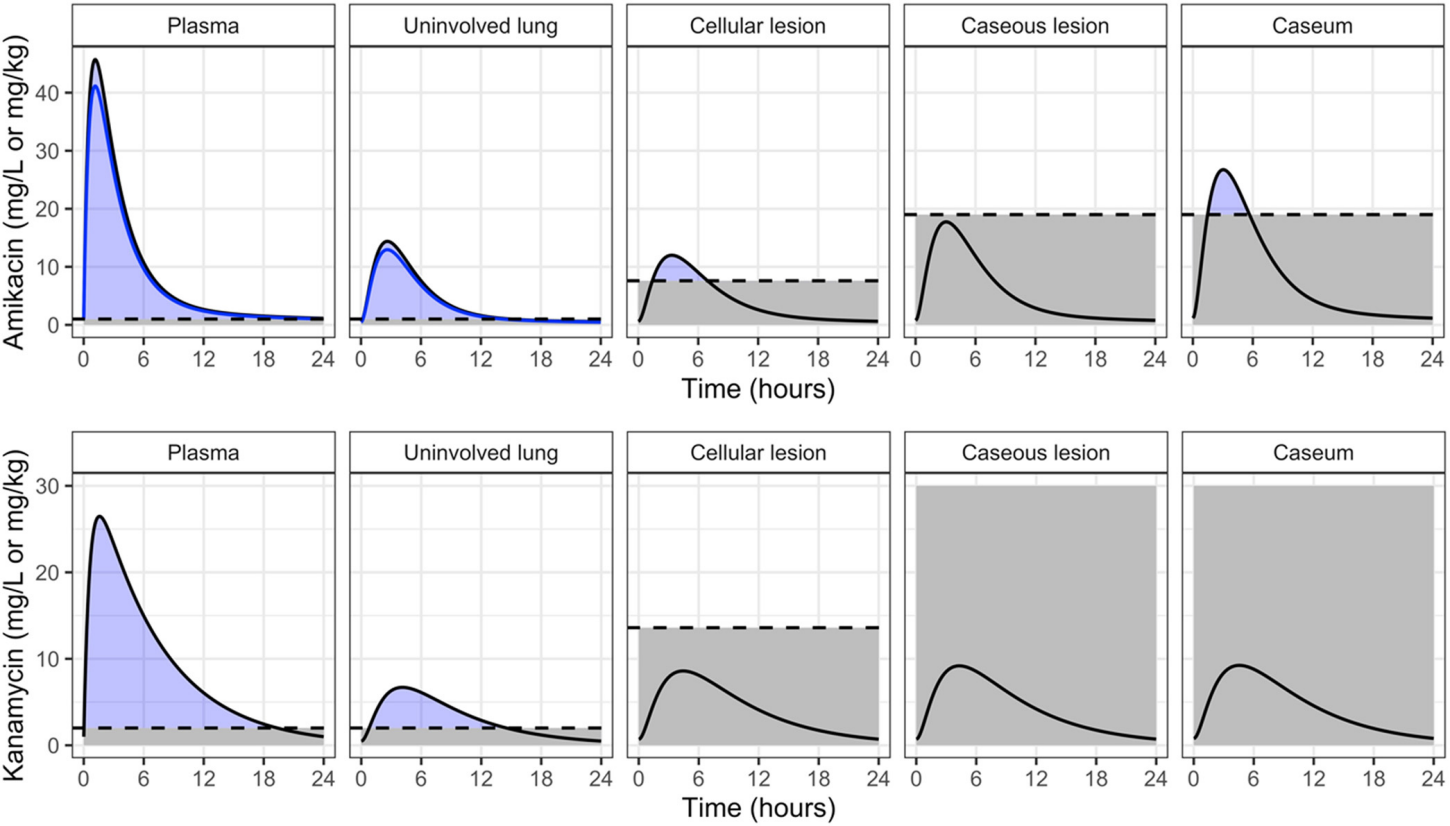
Clinical plasma and site-of-action PK simulations for a 24-h steady-state profile in a typical TB patient. Dashed lines indicate *in vitro* target relevant for each compartment. Plasma and uninvolved lung are relative to MIC (1 mg/liter and 2 mg/liter for AMK and KAN, respectively). Cellular lesion is relative to macrophage IC_90_ (7.6 mg/liter and 13.6 mg/liter for AMK and KAN, respectively). Caseous lesion and caseum are relative to caseum MBC_90_ (19 mg/liter and 248.1 mg/liter for AMK and KAN, respectively). Blue lines for AMK in plasma and uninvolved lung represent the free fraction (KAN plasma protein binding is negligible).

First, lung and lesion penetration coefficients measured in rabbits were applied to clinical plasma concentrations of AMK and KAN to simulate exposure in infected lung compartments. Using published clinical plasma PK models, daily intramuscular injections of 1,000 mg of either AMK or KAN were simulated to steady state (Fig. 5) (35, 49). The ratios between the plasma AUC for the free, unbound drug fraction and the MIC (*f*AUC/MIC) of 294.1 and 108.5 and the *fC*max/MIC of 44.6 and 13 for AMK and KAN, respectively, appeared favorable based on PK-PD studies in patients with Gram-negative and Gram-positive infections (40, 58). In these patient populations, a *C*_max_/MIC ratio of 8 to 10 and a AUC/MIC ratio of 80 to 100 have been associated with effective treatment and prevention of resistance (26, 40, 59). Corresponding thresholds have not been established for TB and would be challenging to validate given the intrinsic multidrug nature of TB treatment. Regardless, the plasma PK-PD parameters of AMK and KAN obtained in our simulations comfortably meet these thresholds. This was true whether total or free drug concentrations in plasma were used since the aminoglycoside free fraction is high.

Next, we applied lesion penetration coefficients measured in rabbits to the clinical plasma PK models then calculated PK-PD parameters in TB lesion compartments using MIC as the denominator (Table S4). While these remained within the desirable range in lesions, their relevance is limited given (i) the disconnect between replicating Mtb drug susceptibility in broth compared to susceptibility of intracellular and extracellular Mtb at the site of disease and (ii) the fact that free drug fraction is unknown in tissues. To overcome this caveat, we assessed PK-PD coverage in lesions using potency values against the two major bacterial populations as measured in the previous section, intracellular 90% inhibitory concentration (IC_90_) in macrophages and MBC_90_ in *ex vivo* caseum (Fig. 5 and Table S4). Unlike MIC, these two assays better reproduce the lesion environment and thus measure free drug potency. In contrast to coverage based on plasma PK and MIC, these simulations revealed poor lesion coverage and poor target attainment at the sites of disease. *C*_max_/MBC_90_ in caseum or caseous lesions was 1.7 for AMK and around 0.04 for KAN. In uninvolved lung and cellular lesions where Mtb is mostly intracellular, AMK reached the intramacrophage IC_90_ for a very short portion of the dosing interval around the *T_max_*, and KAN did not achieve it at all (Fig. 5). Thus, PK-PD parameters that integrate drug concentrations in cellular and caseous TB lesions versus potency against intracellular Mtb and nonreplicating persisters in caseum show that potency targets are not achieved by the second-line aminoglycosides at the site of disease. This may explain their limited contribution to treatment success in MDR-TB patients (3).

## DISCUSSION

The efficacy of second-line injectables AMK and KAN has not been evaluated in placebo-controlled randomized trials that would measure their contribution to MDR-TB regimens. Our knowledge therefore largely relies on meta-analyses that retrospectively assess the benefits of AMK and KAN inclusion in second-line regimens. In the context of newer MDR-TB regimens that include linezolid, bedaquiline, and clofazimine, these analyses indicate that AMK provides modest benefits compared to regimens without injectables, while KAN was associated with worse outcomes (3, 4). Both drugs appeared to have modest benefits in past studies that compared weaker drugs and regimens (55). These meta-analyses also showed that aminoglycosides were associated with the highest incidence of adverse events leading to permanent drug discontinuation (60). However, use of second-line injectables is justified by their potential to prevent emergence of resistance to companion drugs (31, 32).

To help quantify the contribution of AMK and KAN to relapse-free cure and rationalize their use against MDR-TB, we measured and modeled drug concentrations at the site of infection in rabbits with active TB, applied lesion penetration coefficients to clinical plasma PK models, measured concentrations required to kill or inhibit growth of intramacrophage and caseum Mtb, and simulated lesion PK-PD coverage of both agents in key lesion compartments. The simulations show that potency targets are not achieved by KAN in cellular and necrotic lesions and that AMK reaches caseum MBC_90_ for a few hours but with a low caseum *C*_max_/caseum MBC_90_ of 1.7. These results are consistent with the modest benefits of AMK and worse outcomes associated with KAN use (3) and support recent WHO recommendations that “kanamycin be replaced by amikacin, based on evidence from the comparative effectiveness.” (13). SM, which presents (i) similar plasma PKs in patients (22), (ii) similar penetration in rabbit lung lesions, and (iii) the same caseum MBC as AMK, was deemed very weakly bactericidal in cavities in which the great majority of bacilli are present in smear-positive pulmonary TB based on outcomes of an early bactericidal activity trial (61).

Our findings also support WHO recommendations to replace second-line injectables with newly approved agents bedaquiline and delamanid when available, given the irreversible toxicity induced by long-term aminoglycoside use. Interestingly, bedaquiline-containing regimens are more cost effective based on cost-per-treatment success compared with injectable-containing regimens (62). However, recent studies advocate for the maintenance of second-line injectables owing to their acquired resistance-preventing activity (31) and the rising rate of acquired resistance to bedaquiline (30). In plasma and major sites of disease, AMK but not KAN achieved recommended targets of *C*_max_ and AUC relative to MIC. This may translate into controlling emergence of resistance in compartments that are permissive to Mtb replication, where MIC may partially represent drug potency. However, Mtb also replicates intracellularly in the cellular rim of lesions where PK-PD coverage is critically low for AMK and below the threshold across the dosing interval for KAN (Fig. 5). This raises the concern of acquired resistance to these second-line injectables while on therapy (63, 64). Whereas a thrice weekly dosing scheme has been proposed to limit AMK toxicity (8, 30), a study that compared the incidence of ototoxicity and nephrotoxicity in patients receiving either 25 mg/kg thrice weekly or 15 mg/kg daily did not detect any difference in outcome (12). In addition, the thrice weekly dosing scheme may also decrease the already modest lesion coverage despite the long postantibiotic effect of aminoglycosides and further open the door for acquired resistance.

This study has a few limitations. First, a compromise between matching clinical *C*_max_ and AUC in rabbits was adopted due to the high aminoglycoside clearance in rabbits. Because we selected a dose on the higher side of this compromise (∼3-fold higher *C*_max_ and ∼10 to 20% lower AUC in rabbits than in TB patients) and since KAN and AMK do not or barely reach the target potency at this high *C*_max_, putting a stronger emphasis on matching clinical *C*_max_ even if the clearance were artificially decreased to “humanize” the rabbit model would likely reinforce the conclusions of the study. Second, TB patients who received KAN also received multiple first- and second-line drugs. While aminoglycosides are largely excreted unchanged in the urine and thus not prone to metabolic drug-drug interactions, we cannot exclude drug-drug interactions at the level of transport and efflux in and out of tissues, lesions, and immune cells. Third, KAN, but not AMK, was included in the clinical lesion PK study. Because the two drugs display similar physicochemical properties, similar *in vitro* PK parameters known to influence lesion penetration (caseum binding, macrophage uptake, plasma protein binding), and similar plasma and lesion PKs in rabbits, we leveraged the favorable rabbit-to-human translation of the KAN findings to extend the AMK distribution patterns from rabbits to humans. AMK, KAN, and SM all exhibit attractive physicochemical properties that translate into favorable and rapid penetration into necrotic nodules and cavities as well as good retention in nonvascularized cavity caseum. This highlights the potential of the class if one could discover analogs with improved toxicity profiles and potency against nonreplicating persisters. Recent progress with apramycin, a veterinary drug currently in clinical trials, indicates that dissociation between antibacterial activity and ototoxicity is achievable (18, 65). A formulation that maximizes pulmonary delivery relative to systemic drug concentrations and/or promotes slow release to minimize the frequency of injections would further improve their clinical utility. In addition, aminoglycosides were shown to synergize with cell wall-active agents such as β-lactams (66), a class of antibiotics that has generated renewed interest against MDR-TB (67, 68).

In conclusion, the pharmacological profiling of AMK and KAN in pulmonary TB provides an explanation for their limited contribution to MDR-TB regimens and a rationale for the WHO recommendation to replace KAN with AMK. Together with recent mechanistic studies dissecting antibacterial activity from aminoglycoside ototoxicity, the rapid penetration of SM, AMK, and KAN to the sites of TB disease supports the development of analogs with improved efficacy and tolerability.

## MATERIALS AND METHODS

### *In vivo* pharmacokinetics in naive and TB-infected rabbits.

All animal studies were performed in biosafety level 2 and biosafety level 3 (BSL3) facilities and were approved by the Institutional Animal Care and Use Committees of the New Jersey Medical School, Rutgers University, Newark, NJ; Hackensack Meridian Health, NJ; or the National Institute of Allergy and Infection Disease, NIH, Bethesda, MD (LCIM-3). All studies followed the guidelines and basic principles in the United States Public Health Service Policy on Humane Care and Use of Laboratory Animals. All samples collected from M. tuberculosis-infected animals were handled and processed in the BSL3 facility in compliance with protocols approved by the Institutional Biosafety Committee of the New Jersey Medical School, Rutgers University, Newark, NJ, and Hackensack Meridian Health, NJ.

For pharmacokinetic studies in rabbits, female New Zealand White (NZW) rabbits (Charles River Laboratories, Canada), weighing 2.2 to 2.6 kg, were maintained under specific pathogen-free conditions and were fed water and chow *ad libitum*. In dose-finding pharmacokinetic studies in uninfected animals, rabbits received a single dose (60 mg/kg) of AMK formulated in 0.9% saline administered via the intramuscular route.

For plasma and tissue pharmacokinetics in TB-infected animals, NZW rabbits were infected with a high inoculum of M. tuberculosis HN878, using a nose-only aerosol exposure system as described in (69). Approximately 1,000 to 3,000 CFU were recovered from 2 rabbits analyzed 3 h postinfection. At 14 to 20 weeks postinfection, once mature cellular and necrotic lesions had developed, rabbits received three daily doses of AMK or KAN at 25 mg/kg or a single dose of SM (20 mg/kg). Blood was collected from the central ear artery of each rabbit predose and at several time points between drug administration and necropsy (typically 0.5, 1, 2, 4, and 6 h following drug administration until the time of euthanasia). Groups of 3 to 6 rabbits were euthanized 2 h (selected as the earliest time point allowing for distribution from plasma to tissue) and 6 h (the latest practical time point that minimizes the risk of plasma or tissue levels falling below the limit of quantitation) postdose for AMK and KAN. For SM, a single time point was selected at 3 h postdose. Due to the rapid clearance of AMK and KAN and the analytically challenging nature of the aminoglycosides, plasma and lesions collected at 24 h postdose could have delivered a large percentage of BLOQ (below the limit of quantitation) data points. All blood samples were centrifuged at 4,000 rpm for 5 min, and the supernatants (plasma) were transferred and stored at −80°C until analyzed by high-pressure liquid chromatography coupled to tandem mass spectrometry (LC/MS-MS).

### Collection of human tubercular tissues.

Patients undergoing elective resection surgery to debulk MDR or XDR M. tuberculosis-infected lung segments were recruited into a previously reported multicenter clinical study (NCT00816426 [48, 49]) with written consent. The institutional review boards of the National Institute of Allergy and Infection Disease, National Institutes of Health, Bethesda, MD, USA, and the Asan Medical Center, Seoul, Republic of Korea, approved the study. All procedures were in accordance with the ethical standards of the Helsinki Declaration. The patients received a single dose of 1,000 mg KAN if the drug was not part of their current drug regimen at specific times prior to surgery and subsequent tissue removal. During surgery, the exact time of pulmonary artery ligation was recorded and used to calculate the time of drug administration relative to surgery (Table S2 in the supplemental material). Following lung resection, the tissue was immediately dissected into individual tubercular lesions that were snap frozen in liquid nitrogen vapor for sectioning and laser capture microdissection and stored at −80°C until analyzed.

### Lesion dissection and processing.

From each lung lobe, individual granulomas, mediastinal lymph nodes, and uninvolved (nondiseased) lung tissue areas were dissected, sized, weighed, and recorded. Special care was taken to remove the uninvolved lung tissue surrounding each granuloma. The samples were classified as lymph node, uninvolved lung (devoid of macroscopically visible lesions, although known to be infiltrated with various immune cell types and may contain cellular microlesions), cellular granuloma (appears opaque and feels hard to the touch), necrotic granuloma (shows a white/yellow center under the cellular rim and feels soft to the touch), or cavity (presents a large open and opaque ring with a central air pocket and variable amounts of caseum remaining). When necrotic granulomas were greater than 7 mm, they were dissected so that the lesion wall and the caseous material within could be stored and analyzed separately. Lesions collected for laser-capture microdissection and MALDI mass spectrometry imaging were left embedded in the surrounding tissue and were snap frozen in liquid nitrogen vapor as described previously (47). All samples were stored in individual 2-ml tubes at −80°C.

Prior to drug quantitation by LC-MS/MS, all tissue samples were homogenized in approximately, but accurately recorded, 5 volumes of phosphate-buffered saline (PBS). Homogenization of tissue samples was achieved using a FastPrep-24 instrument (MP Biomedicals) and 1.4-mm zirconium oxide beads (Precellys). Lung and lesion homogenates were stored at −80°C prior to KAN, AMK, and SM quantitation by LC-MS/MS analysis.

### *In vitro* pharmacokinetic assays.

*Caseum-binding assay*. The caseum-binding assay was performed by rapid equilibrium dialysis using a disposable rapid equilibrium dialysis (RED) device (ThermoFisher Scientific, MA) as previously described (50, 70). Briefly, caseum was diluted 10-fold in PBS, homogenized, and spiked at a final incubation concentration of 5 μM. Spiked matrix (200 μl) was placed in the sample chambers, and the buffer chambers were filled with 350 μl of PBS. The plates were then covered with adhesive seals and incubated at 37°C for 4 h on an orbital shaker set at 300 rpm. Following incubation, samples were removed from both chambers and extracted with water containing 33% trichloroacetic acid before LC-MS/MS quantitation. The fraction unbound (*f*_u_) in diluted caseum was calculated as the ratio between free (buffer chamber) and total (sample chamber) drug concentrations.

*Macrophage uptake assay*. Aminoglycoside uptake assays in THP-1 cells were performed as previously reported (71). Briefly, THP-1 cells (ATCC, TIB-202), grown in RPMI 1640 medium supplemented with 10% fetal bovine serum and 2 mM l-glutamine in a CO_2_ incubator, were seeded into wells of a 96-well tissue culture-treated plate at 5 × 10^4^ cells/well. THP-1 monocytes were differentiated overnight to macrophages with 100 nM phorbol 12-myristate 13-acetate (PMA). Culture medium was carefully removed, and medium containing 5 μM AMK, KAN, or SM was added. After 30 min at 37°C, the cells were gently washed twice with ice-cold PBS to remove extracellular drug. Cells were lysed with deionized water for 1 h at 37°C. The drug content of cell lysates was analyzed by LC/MS-MS and subsequently normalized to (i) the number of cells per well after drug treatment and washing of dead (nonadherent) cells if any and (ii) the average cellular volume to calculate the intracellular concentration of each aminoglycoside. The drug accumulation factor is expressed as a ratio between the intracellular concentration and extracellular concentration (IC/EC).

### *In vitro* pharmacodynamic assays.

*Intracellular*M. tuberculosis*potency assay in THP-1-derived macrophages*. To measure aminoglycoside activity against intracellular bacteria, THP-1 monocytes were cultured as mentioned above and differentiated to macrophages with PMA on 24-well cell culture-treated plates seeded with 5 × 10^5^ cells/well. The macrophages were infected with the Erdman strain of M. tuberculosis at a multiplicity of infection of 1:1. After 4 h of infection, the wells were washed three times with PBS to remove extracellular bacteria. Fresh medium containing 1, 5, 20, 50, or 100 μM of each study drug was added, with vehicle-only wells included as controls. After 1, 2, and 3 days at 37°C and 5% CO_2_, the THP-1 macrophages were detached with 5 mM EDTA and lysed with 0.05% SDS, and serial dilutions of the lysates were plated on Middlebrook 7H11 agar for CFU enumeration.

*Caseum minimum bactericidal concentration assay*. The minimum bactericidal concentration assay against M. tuberculosis found in rabbit caseum (MBC_90_ in *ex vivo* caseum) was performed as described previously (53). Briefly, rabbit caseum was homogenized and incubated with SM, AMK, or KAN at concentrations ranging from 0.03125 to 512 μM for 7 days and was then plated on Middlebrook 7H11 agar for CFU enumeration, including no-drug controls. The MBC in caseum, or casMBC_90_, is defined as the minimum concentration that kills 90% of endogenous bacteria residing in caseum.

### Analytical methods for quantitation and imaging of AMK, KAN, and SM.

*LC-MS/MS method for quantitation of aminoglycosides in plasma and tissue homogenates*. KAN, AMK, and SM internal standards were purchased from Sigma-Aldrich. Drug-free K_2_EDTA plasma and lungs from NZW rabbits were obtained from BioIVT for use as blank matrices to build standard curves. Neat 1 mg/ml Milli-Q stocks were serially diluted in water containing 1% formic acid (FA) to create neat standards. Control tissue and study sample homogenates were created by adding 9 parts of PBS buffer to 1 part of tissue (10× dilution) and shaking the samples using a Fisher bead mill for 1 min at 6,000 × *g* with zirconia beads. Standard, quality control, and study samples were extracted by combining 10 μl of tissue homogenate or plasma, 10 μl of 500 ng/ml internal standard, and 100 μl of water containing 33% trichloroacetic acid. AMK was used as an internal standard for SM sample analysis, and SM was used for AMK and KAN sample analysis. Extracts were vortexed for 5 min and centrifuged at 4,000 rpm for 5 min. Supernatant (100 μl) was transferred to a 96-well plate for LC-MS/MS analysis. LC-MS/MS analysis was performed on a Sciex Applied Biosystems Qtrap 6500+ triple quadrupole mass spectrometer coupled to a Shimadzu Nexera X2 UHPLC system to quantify each drug in plasma. Chromatography was performed on an Agilent Zorbax SB-C_8_ column (2.1 × 30 mm; particle size, 3.5 μm) using a reverse-phase gradient elution with aqueous. Milli-Q deionized water with 0.1% FA and 0.1% heptafluorobutyric acid (HFBA) was used for the aqueous mobile phase and 0.1% FA and 0.1% HFBA in acetonitrile for the organic mobile phase. Representative chromatograms are shown in Fig. S5. Multiple-reaction monitoring (MRM) of precursor/fragment transitions in electrospray positive-ionization mode was used to quantify the analytes. MRM transitions of 586.70/163.20, 485.40/163.00, and 582.30/263.30 were used for AMK, KAN, and SM, respectively. Sample analysis was accepted if the concentrations of the quality control samples were within 20% of the nominal concentration. Data processing was performed using Analyst software (version 1.6.2; Applied Biosystems Sciex).

*Laser-capture microdissection*. LCM was performed as previously described (47). Briefly, γ-irradiated frozen lung biopsy specimens were sectioned at 10 μm for histology and 25 μm for LCM using a CM1810 cryostat (Leica). Sections for histological analysis were taken immediately adjacent to those taken for LCM, and data were correlated. LCM sections were thaw mounted onto 1.4-μm-thick polyethylene terephthalate (PET) membrane slides (Leica). Regions of necrotic caseum, their corresponding cellular rim, and normal lung tissue were dissected using an LMD7 scope (Leica) until an area of 3 million μm^2^ had been collected for each region. Dissected regions of interest were stored at −80°C until analysis.

*LC-MS/MS method for quantitation of AMK and KAN in laser capture microdissected samples*. LCM sample quantification was performed according to a previously published protocol (47). Briefly, neat 1 mg/ml Milli-Q stocks of AMK and KAN were serially diluted in water containing 1% formic acid to create neat standards. Control tissue homogenate was created by adding 25.6 parts PBS buffer to 1 part tissue (26.7× dilution) and shaking the samples using a Fisher bead mill for 1 min at 6,000 rpm with zirconia beads. Standard, quality control, and control samples were extracted by adding 2 μl of blank homogenate, 10 μl of neat standard, 5 μl of 500 ng/ml SM as internal standard, and 50 μl of water containing 33% trichloroacetic acid. LCM study samples were extracted identical to standards using 2 μl of PBS in place of tissue homogenate. Extracts were bath sonicated for 10 min and centrifuged at 4,000 rpm for 5 min. Supernatant (50 μl) was transferred to a 96-well plate for high-performance liquid chromatography (HPLC)-MS/MS analysis. HPLC-MS/MS analysis was performed as described in the whole tissue analysis methods.

### MALDI mass spectrometry imaging.

*Sample preparation and data acquisition*. Mass spectrometry imaging experiments were performed on 10-μm sections taken adjacent to those used for histological registration of the LCM sections, as described above. Tissue washing was performed before matrix deposition by submerging the slides in chloroform for 15 s at −20°C as previously published (72). The slides were then allowed to air dry for 10 min before matrix deposition. The 2,5-dihydroxybenzoic acid (DHB) matrix solution (20 mg/ml in 50% methanol, 5 mM sodium chloride, and 0.1% trifluoroacetic acid) was deposited over the tissue sections using the HTX M5 sprayer (HTX Technologies LLC, Chapel Hill, NC, USA) using the following parameters: 60°C nozzle temperature, 50 μl/min flow rate, 900 velocity, 8 lb/in^2^ 26 passes, and crisscross spray pattern. Data acquisition was carried out using a Bruker solariX 7 Tesla FT-ICR mass spectrometer (Bruker Daltonics, Billerica, MA, USA) equipped with a dual electrospray ionization (ESI)/MALDI ion source and Smartbeam II Nd:YAG (355-nm) laser. The instrument was operated in the positive ion mode within the mass range of *m/z* 150 to 3,000, utilizing the continuous accumulation of selected ions (CASI) function in which the quadrupole mass was set to an *m/z* of 550 with an isolation window of 200. Images were acquired at 50-μm resolution using the small laser setting and 200 laser shots per pixel. The transient length was 0.7340, which resulted in an estimated resolving power of 99,000 at an *m/z* of 400 (full width at half maximum). Following data acquisition, the slides were washed with 70% ethanol to remove the matrix and stained with hematoxylin and eosin (H&E). The stained sections were then coregistered with the MSI data for analysis.

*Data analysis*. Data analysis was performed using the SCiLS Lab MVS, version 2020a Pro (Bruker Daltonics, Billerica, MA, USA). The data files for the 2-h and 6-h postdose time points were combined and imported into a single file for each drug compound to enable comparison across time points and for statistical analysis. MS images of KAN and AMK were processed using the weak denoising function and were presented using the rainbow scale color scheme. Regions of interest (ROIs) were drawn around the different histologically identifiable areas of TB-infected lung tissue; these included inner and outer caseum, the cellular layer, and normal tissue lung parenchyma. From each of these regions ∼350 spectra were acquired from individual pixels (50 × 50 μm) used to create relative pixel intensity plots of KAN and AMK ion abundance in each region. The spectral data obtained from the polygonal ROIs in SCiLS Lab were exported and further analyzed in GraphPad Prism (GraphPad Software, San Diego, CA, USA).

### Pharmacokinetic-pharmacodynamic modeling and simulations.

Data from a single dose of AMK (60 mg/kg) in uninfected rabbits were used to develop a plasma PK model. Simulations were performed to match exposure in rabbits predicted to be equivalent to a 1,000 mg dose in humans. To describe the movement of drug from plasma to sites of action, nonlinear mixed effects models were built for KAN and AMK using available rabbit data (Table S1). Tissue density was assumed to be 1 g/ml of homogenate. Plasma and lesions were modeled sequentially, where plasma data were modeled first. The plasma parameters were fixed, and effect compartments were added for each distinct tissue. Samples quantified by both homogenate/LC-MS and LCM/LC-MS methods were pooled and treated equally. A rate (*k*_pl-lesion_), ratio (*PC*_pl-lesion_), and residual error were estimated for each lesion type. Model building was guided by goodness-of-fit plots, objective function value, and visual predictive checks. One thousand simulations using interindividual variability and residual error as variability were performed to confirm model fit to raw data. NONMEM version 7.4.2, R software version 3.6.1, and the R packages ggplot2, xpose4, and PKPDsim were used for model building, data visualization, and simulations. A translational model was developed by linking lesion parameters estimated in rabbits to previously published clinical plasma PK models (35, 49). Clinical simulations were compared to *in vitro* targets, and unbound *C*_max_ and AUC and time relative to *in vitro* targets were quantified. Fraction unbound was assumed to be >0.99 for KAN (73) and 0.9 for AMK (74). Model structure and model diagnostic plots are shown in Fig. 3.
