# Supplementary material for: Lesion Penetration and Activity Limit the Utility of Second-Line Injectable Agents in Pulmonary Tuberculosis
Source: Antimicrob Agents Chemother. 2021 Sep 17;65(10):e00506-21. doi: 10.1128/AAC.00506-21 (PMC8448094; doi:10.1128/AAC.00506-21)
Supplement: Supplemental file 1 — Supplemental tables and figures. Download AAC.00506-21-s0001.pdf, PDF file, 0.5 MB [file aac.00506-21-s0001.pdf]

Supplemental Tables

Table S1. Comparative pharmacokinetic parameters of AMK and KAN at 1,000 mg in human subjects and 25 mg/kg in New Zealand White rabbits.

	**AMK**	**KAN**
C_{max} rabbits 25 mg/kg (mg/L)	90 (75 – 110)	101 (75-133)
C_{max} clinical (1,000 mg or 15 mg/kg) (mg.h/L)	33 (20 – 60) (1-3)	35 (20 – 50) (4)
AUC rabbits 25 mg/kg (mg/L)	178 (140 – 250)	178 (147 – 213)
AUC clinical (1,000 mg or 15 mg/kg) (mg.h/L)	225 (5, 6)	190 (7)

Table S2. Description of human subject characteristics (ClinicalTrials.gov ID #NCT00816426)

Subject	Gender	Age	BMI	Weight (kg)	Prior TB episodes [(a)]	Study drugs single dose	Study drugs at steady state	Cavitary disease	Other anti-TB agents at steady state[(b)]	time of surgery post study drugs
G101	Male	27	24.0	73.7	2	INH, RIF, PZA	MFX, KAN	Yes	LZD, AUG, CLA	25h 20min
G402	Male	40	17.3	57	2	RIF, PZA, KAN	INH, MXF	Yes	EMB	24h 20min
G105	Male	54	29.4	84	2	INH, RIF, PZA, MXF, KAN		Yes	LZD, AMK, PAS, CFZ, PIP	12h 35min
G106	Female	48	22.2	54.3	1	INH, RIF, PZA, KAN	MFX	Yes	LZD, CS, AMX, PTH, PAS	11h 00min
G102	Male	43	27.1	79.6	2	INH, RIF, PZA	MFX, KAN	Yes	LZD, PAS, AUG	11h 12min
G103	Female	23	18.8	49.8	2	INH, RIF, PZA, MXF, KAN		No	LZD, CS, CFZ	4h 56min
G108	Male	47	18.9	57.8	1	RIF, PZA, MXF, KAN	INH	No	LZD, CS, AUG, PTH, STM	5h 41min
G303	Male	39	22.2	65.7	4	INH, RIF, MXF, KAN	PZA	Yes	CS, LFX, PTH, STM	4h 23min
G109	Female	58	21.2	62	1	INH, RIF, PZA, MXF	KAN	?	LZD, CS, PAS	21h 55min
G104	Female	27	20.0	52.5	1	INH, RIF, MXF, KAN	PZA	No	LZD, CS, AUG, STM	3h 18min
G401	Male	44	24.1	58	1	RIF, PMXF, KAN	INH, PZA	Yes	EMB, LFX	8h 30min

[(a)] all subjects had either MDR- or XDR-TB

[(b)] AMK, amikacin; AUG, amoxicillin/clavulanate; CFZ, clofazimine; CLA, clarithromycin; CS, cycloserine; EMB, ethambutol; LFX, levofloxacin; LZD, linezolid; PAS, para-aminosalicylate; PTH, prothionamide; STM, streptomycin; PIP: piperacillin/tazobactam.

Table S3. Number of observations of KAN or AMK concentration in rabbit plasma and tissue including uninvolved lung and tubercular lesions. Samples were analyzed via LCMS or LCM. n, number of samples; N, number of subjects, LCMS, liquid chromatography mass spectrometry; LCM, laser capture microdissection.

	**AMK**	**KAN**
Observations, total (n, N)	169, 9	144, 5
Plasma (LCMS, LCM)	45, 0	26, 0
Uninvolved lung (LCMS, LCM)	23, 11	29, 12
Cellular lesions (LCMS, LCM)	9, 11	26, 12
Caseous lesions (LCMS, LCM)	59, 0	24, 0
Caseum (LCMS, LCM)	0, 11	1, 14

Table S4. Model-based clinical predictions of C_{max}, AUC, and time relative to MIC per lesion for (A.) AMK and (B.) KAN. C_{max} and unbound C_{max} relative to MIC, MacIC90, and casMBC90. Values in mg/L (plasma) or mg/kg (lung and lesions). AUC and unbound AUC relative to MIC. Values in mg*h/L (plasma) or mg*h/kg (lung and lesions). Time above MIC, Macrophage IC_{90}, or caseum MCB_{90} within a 24-hour period of once daily dosing at steady-state. Values are hours above target with a maximum of 24.

A. AMK

	Plasma	Uninvolved lung	Cellular lesion	Caseous lesion	Caseum
Cmax	45.7	14.4	12.0	17.7	26.7
fCmax	41.1	12.9	10.8	16.0	24.1
fCmax/MIC	41.1	12.9	10.8	16.0	24.1
Cmax/MacIC90	6.0	1.9	1.6	2.3	3.5
Cmax/casMBC90	2.4	0.8	0.6	0.9	1.4
AUC	216.9	94.8	100.2	134.1	201.1
fAUC/MIC	195.2	85.3	90.2	120.7	181.0
Time above MIC	24	14	17	19	24
Time above MacIC90	7	6	5	7	9
Time above casMBC90	4	0	0	0	4

B. KAN

	Plasma	Uninvolved lung	Cellular lesion	Caseous lesion	Caseum
Cmax	26.5	6.7	8.6	9.2	9.2
fCmax	26.5	6.7	8.6	9.2	9.2
fCmax/MIC	13.2	3.4	4.3	4.6	4.6
Cmax/MacIC90	1.9	0.5	0.6	0.7	0.7
Cmax/casMBC90	0.1	0.0	0.0	0.0	0.0
AUC	216.9	73.3	98.5	103.3	107.8
fAUC/MIC	108.5	36.7	49.2	51.6	53.9
Time above MIC	19	14	16	17	17
Time above MacIC90	6	0	0	0	0
Time above casMBC90	0	0	0	0	0

Supplemental Figures

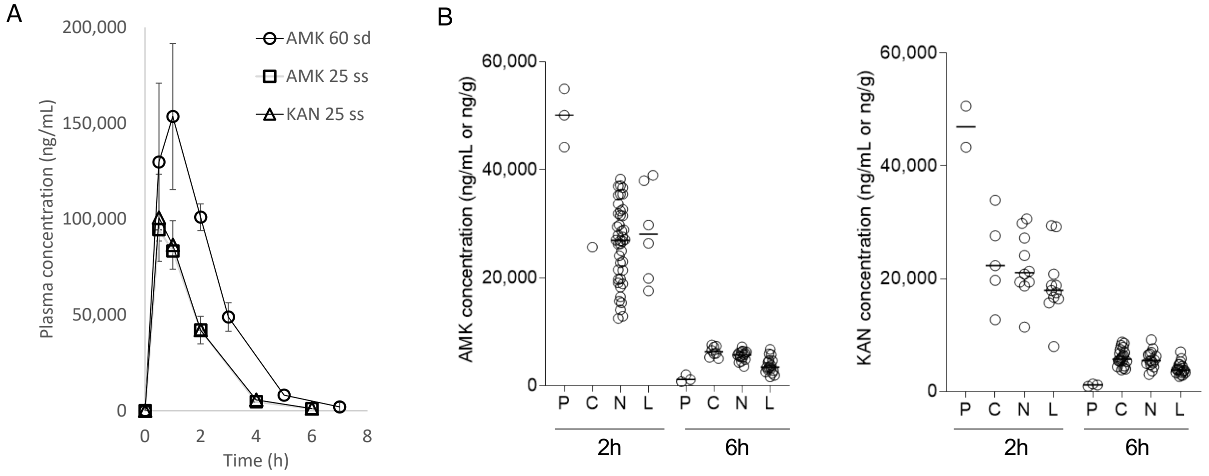

Figure S1. Rabbit pharmacokinetics of AMK and KAN. **(A)** Plasma concentration-time profiles in uninfected rabbits and rabbits with active TB. In a dose finding study, three uninfected rabbits received a single 60 mg/kg dose (sd) of AMK administered via the intramuscular route; in tissue and lesion distribution studies, groups of 5 or 6 rabbits received 3 daily doses (steady state or ss) of AMK or KAN administered via the intramuscular route, as indicated. **(B)** Drug concentrations in lung and lesion homogenates of TB infected rabbits following three daily doses of 25 mg/kg AMK and KAN. P: plasma; C: cellular lesion; N: necrotic (caseous) lesion; L: uninvolved lung. A sample size of 3 animals per drug treatment and time point was selected based on historic ability to build PK models that deliver adequate fit. Animals that did not present adequate pathology or adequate number of evaluable lesions were replaced until we reached N=3. For LCMS quantitation in homogenized tissue/lesions, the following number of lesions per animal were collected: (i) 6 pieces of uninvolved lung with the exception of 2 rabbits at the 2h AMK time point, which had extensive pathology to the extent that it was difficult to locate uninvolved lung (3 pieces were collected in these rabbits), (ii) 5 to 10 cellular lesions with the exception of the same 2 rabbits where all but one lesion were necrotic, (iii) 3 to 26 necrotic lesions (more necrotic lesions were collected when cellular lesions could not be found).

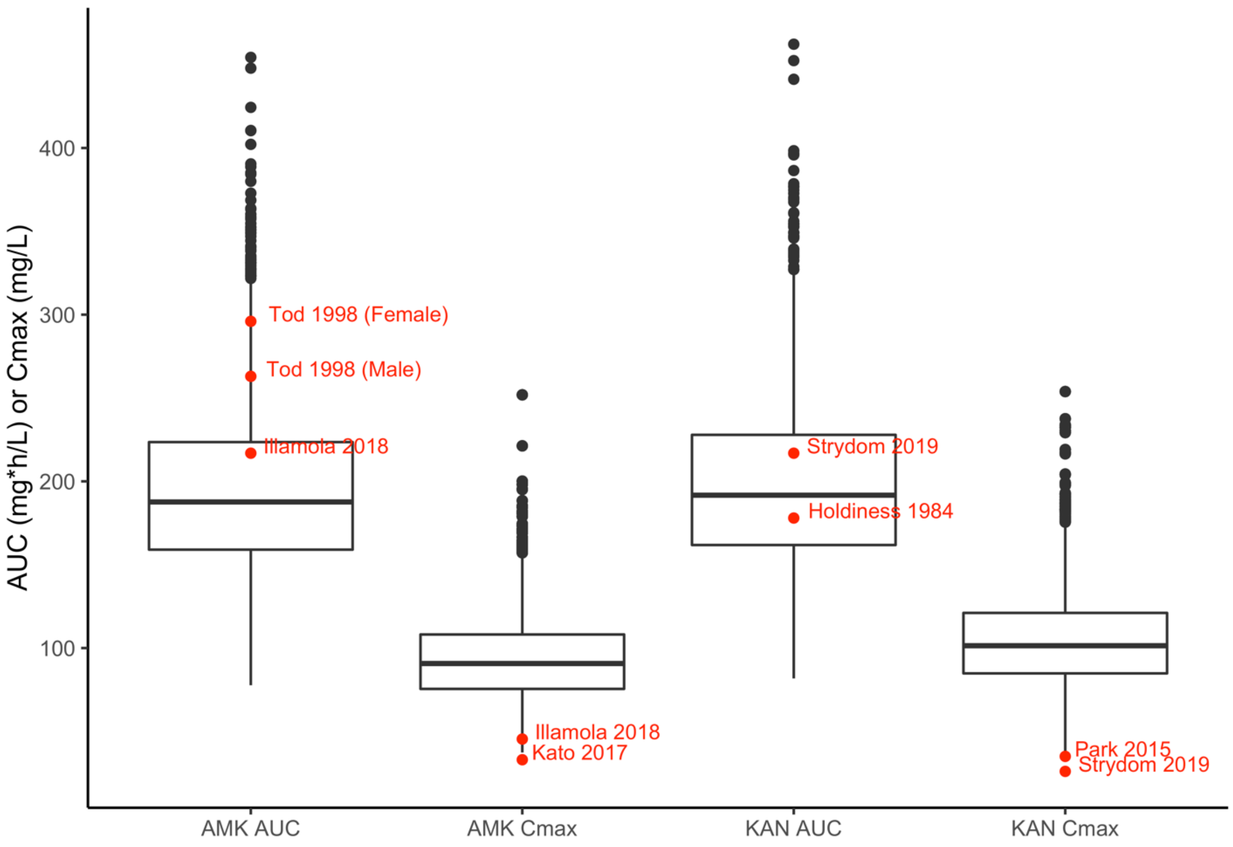

Figure S2. Comparison of steady state AUC (area under the concentration-time curve) and C_{max} (peak plasma concentration) in rabbits after three 25 mg/kg intramuscular doses (black box and whisker plots) and in TB patients receiving 1,000 mg daily (red dots retrieved from published studies). Emphasis was placed on matching AUC since it is considered the driver of aminoglycoside efficacy. References: (1, 4, 5, 7-9)

A

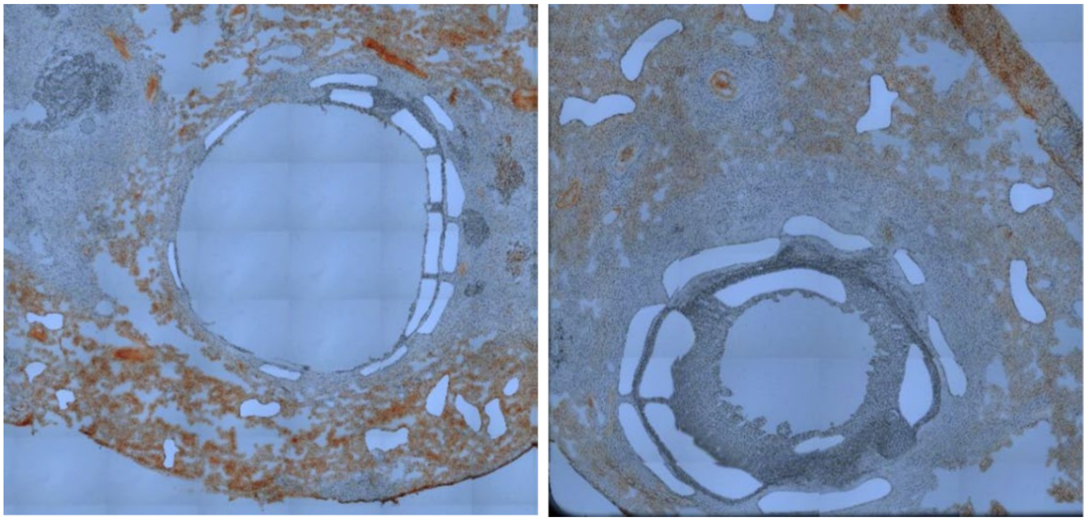

B

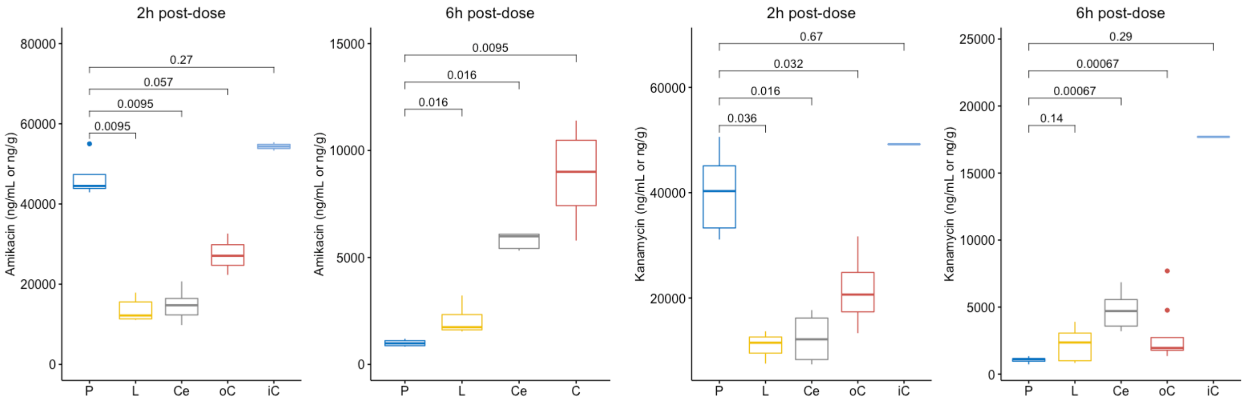

Figure S3. Sample size and statistical analysis of data shown in **Figure 1C**. For drug quantitation by laser capture microdissection, large necrotic lesions and cavities were collected with the surrounding uninvolved lung, as follows: AMK 2h: 6 lesions; AMK 6h: 6 lesions; KAN 2h: 4 lesions; KAN 6h: 8 lesions. **(A)** Representative large cavities collected from the AMK rabbits analyzed 6h post dose, from which the inner caseum is missing, either because it emptied prior to lesion removal at the time of euthanasia, or because it fell apart at the time of cryosectioning. Consequently, only “caseum” (C) is reported in Figure 1C for AMK at 6h post dose. **(B)** Box-and-whisker plots and statistical analysis of absolute concentrations of AMK and KAN in plasma and infected lung regions determined by laser-capture microdissection and LC/MS-MS. P: plasma; L: uninvolved lung; Ce: cellular rim; C: caseum; oC: outer caseum; iC: inner caseum.

The horizontal bar is the median, the hinges are the interquartile range (IQR), the whiskers extend to 1.5*IQR, and dots are outliers. Drug concentrations in tissue compartments were compared to plasma concentrations using the Wilcoxon test.

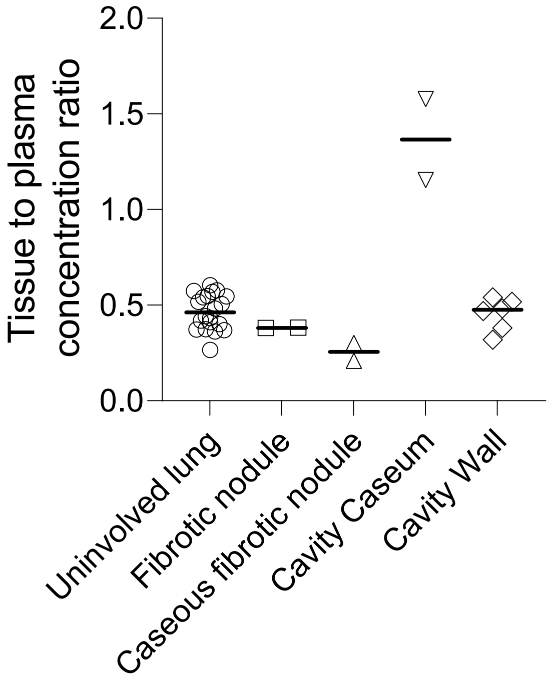

Figure S4. Partitioning of streptomycin in rabbit lung and lesion compartments 3h after a single 20 mg/kg dose. Concentrations were measured in tissue homogenates and normalized to plasma concentrations at the time of necropsy.

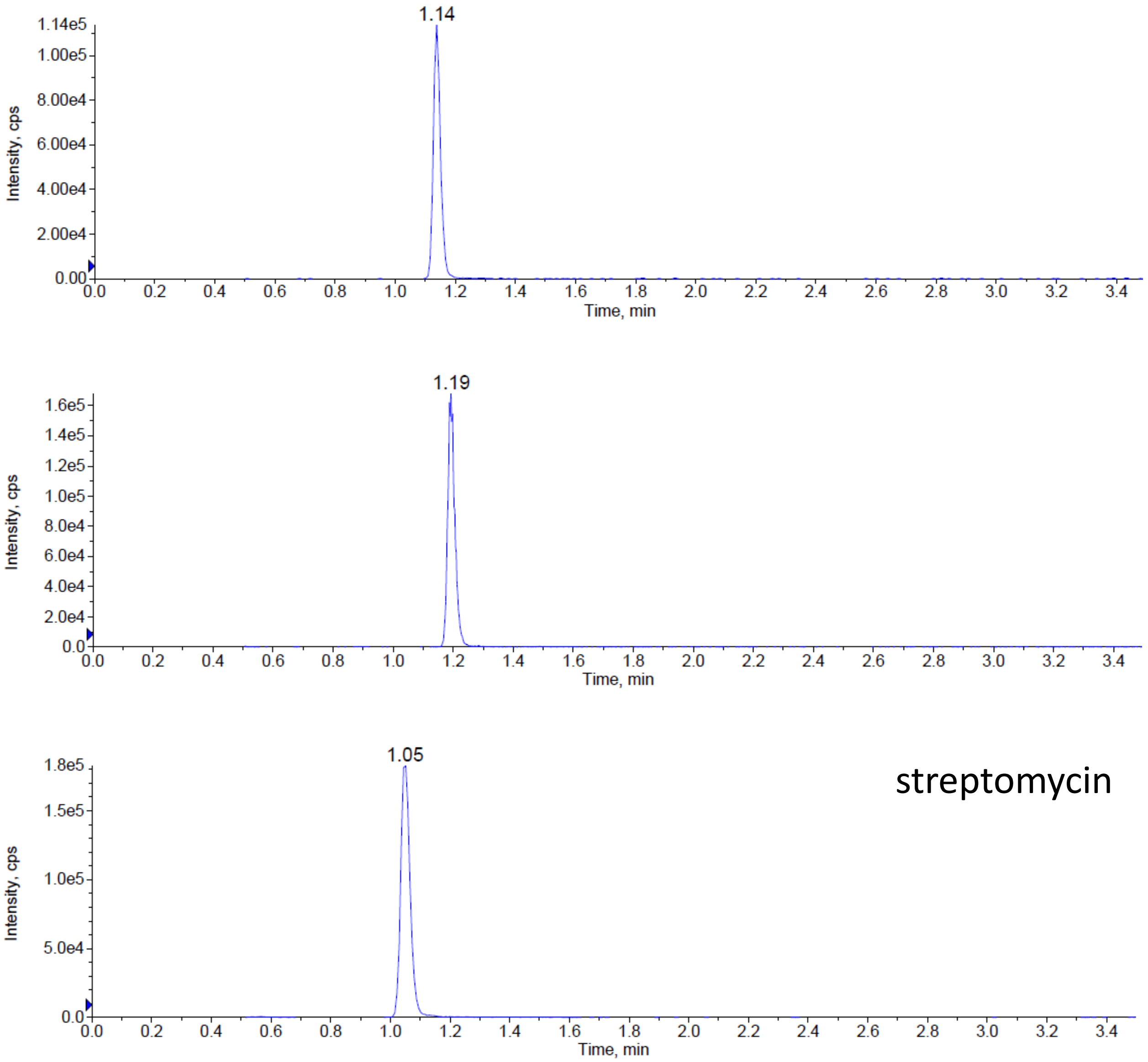

Figure S5. Typical plasma standard HPLC-MS/MS chromatograms for amikacin, kanamycin, and the internal standard streptomycin, at 5000 ng/mL.

REFERENCES

1. Kato H, Hagihara M, Hirai J, Sakanashi D, Suematsu H, Nishiyama N, Koizumi Y, Yamagishi Y, Matsuura K, Mikamo H. 2017. Evaluation of Amikacin Pharmacokinetics and Pharmacodynamics for Optimal Initial Dosing Regimen. Drugs R D 17:177-187.
2. Sadeghi K, Hamishehkar H, Najmeddin F, Ahmadi A, Hazrati E, Honarmand H, Mojtahedzadeh M. 2018. High-dose amikacin for achieving serum target levels in critically ill elderly patients. Infect Drug Resist 11:223-228.
3. Arechiga-Alvarado NA, Medellin-Garibay SE, Milan-Segovia RDC, Ortiz-Alvarez A, Magana-Aquino M, Romano-Moreno S. 2020. Population Pharmacokinetics of Amikacin Administered Once Daily in Patients with Different Renal Functions. Antimicrob Agents Chemother 64.
4. Park SI, Oh J, Jang K, Yoon J, Moon SJ, Park JS, Lee JH, Song J, Jang IJ, Yu KS, Chung JY. 2015. Pharmacokinetics of Second-Line Antituberculosis Drugs after Multiple Administrations in Healthy Volunteers. Antimicrob Agents Chemother 59:4429-35.
5. Tod M, Lortholary O, Seytre D, Semaoun R, Uzzan B, Guillevin L, Casassus P, Petitjean O. 1998. Population pharmacokinetic study of amikacin administered once or twice daily to febrile, severely neutropenic adults. Antimicrob Agents Chemother 42:849-56.
6. Yew WW, Cheung SW, Chau CH, Chan CY, Leung CK, Cheng AF, Wong CF. 1999. Serum pharmacokinetics of antimycobacterial drugs in patients with multidrug-resistant tuberculosis during therapy. Int J Clin Pharmacol Res 19:65-71.
7. Holdiness MR. 1984. Clinical pharmacokinetics of the antituberculosis drugs. Clin Pharmacokinet 9:511-44.
8. Illamola SM, Huynh HQ, Liu X, Bhakta ZN, Sherwin CM, Liou TG, Carveth H, Young DC. 2018. Population Pharmacokinetics of Amikacin in Adult Patients with Cystic Fibrosis. Antimicrob Agents Chemother 62.
9. Strydom N, Gupta SV, Fox WS, Via LE, Bang H, Lee M, Eum S, Shim T, Barry CE, 3rd, Zimmerman M, Dartois V, Savic RM. 2019. Tuberculosis drugs' distribution and emergence of resistance in patient's lung lesions: A mechanistic model and tool for regimen and dose optimization. PLoS Med 16:e1002773.
